# Single cell and single nucleus RNA sequencing in liver tissues: applications and prospects in model and non-model organisms

**DOI:** 10.3389/fgene.2026.1781941

**Published:** 2026-04-28

**Authors:** Xue Feng, Yu Feng, Sayed Haidar Abbas Raza, Yun Ma, Hongyu Deng

**Affiliations:** 1 College of Animal Science and Technology, Henan University of Animal Husbandry and Economy, Zhengzhou, China; 2 Department of Pathology, The Seventh People’s Hospital of Zhengzhou, Zhengzhou, China; 3 State Key Laboratory of Biocontrol, School of Life Sciences, Sun Yat-Sen University, Guangzhou, Guangdong, China; 4 Key Laboratory of Ruminant Molecular and Cellular Breeding of Ningxia Hui Autonomous Region, College of Animal Science and Technology, Ningxia University, Yinchuan, China; 5 Henan Key Laboratory of Healthy Breeding and Efficient Reproduction of Livestock and Poultry, Zhengzhou, China

**Keywords:** heterogeneity, liver, pathophysiological, scRNA-seq, snRNA-seq

## Abstract

The liver serves as a central metabolic hub, essential for homeostasis, detoxification, and immunity. Recent advances in single-cell and single-nucleus RNA sequencing (scRNA-seq and snRNA-seq) have fundamentally transformed our capacity to resolve the cellular architecture and functional states of this complex organ. This review comprehensively examines the pivotal applications and expanding potential of these high-resolution transcriptomic technologies in hepatic research, encompassing both established model organisms and emerging non-model species. In human and classical models such as mice and zebrafish, scRNA-seq and snRNA-seq have been critical for delineating developmental trajectories, deciphering the molecular logic of metabolic zonation, and uncovering the precise cellular dynamics and intercellular communication networks that drive diseases like non-alcoholic fatty liver disease (NAFLD), fibrosis, and hepatocellular carcinoma. Beyond these systems, pioneering work in species such as cattle, pigs, tree shrews, and reptiles is now providing unique insights into evolutionary adaptations, specialized physiologies, and comparative disease mechanisms. By synthesizing findings across this broad biological spectrum, we illustrate how single-cell transcriptomics is refining the core principles of liver biology while simultaneously revealing species-specific divergences. Looking ahead, the continued maturation and application of these technologies are poised to yield deeper comparative pathophysiological understanding and accelerate the development of targeted diagnostic and therapeutic strategies for liver diseases in both human and veterinary contexts.

## 1 Introduction

The liver’s remarkable capacity to manage diverse physiological (MacParland et al., 2018; Aizarani et al., 2019) and pathological (Armingol et al., 2021; Massalha et al., 2020) processes is fundamentally rooted in its intricate cellular composition and highly organized architecture. Anatomically composed of repeating hexagonal hepatic lobules (Ben-Moshe and Itzkovitz, 2019), each unit is centered on a portal triad (portal vein, bile duct, hepatic artery) and radiates outward with hepatocyte cords enmeshed in a sinusoidal capillary network, converging at a central vein. This structure establishes a directional flow: nutrient- and toxin-rich blood from the portal vein and oxygenated blood from the hepatic artery mix in the sinusoids, percolate toward the central vein, and eventually drain into systemic circulation, while bile flows inversely from pericentral hepatocytes outward to the bile ducts. This counter-current system creates a longitudinal portal-central gradient of oxygen and nutrients, driving the spatial compartmentalization of metabolic functions—a principle known as liver zonation. Rappaport originally delineated the human liver acinus into periportal (Zone 1), middle (Zone 2), and central vein (Zone 3) zones (Rappaport, 1958; Rappaport, 1973; Rappaport, 1976). Halpern et al. later refined this model using scRNA-seq, proposing a more detailed subdivision of the mouse lobule into nine concentric layers, each exhibiting distinct transcriptional and metabolic specializations (Halpern et al., 2017; Jungermann and Katz, 1982). For example, periportal hepatocytes preferentially express genes for gluconeogenesis, cholesterol synthesis, and ureagenesis (Gebhardt and Matz-Soja, 2014; Jungermann, 1988; Paris and Henderson, 2022), whereas central vein hepatocytes specialize in glycolysis, glutamine synthesis, and bile acid production (Paris and Henderson, 2022). The middle zone has further been identified as a key source of new hepatocytes during homeostasis and regeneration (Wei et al., 2021). Thus, the liver’s sophisticated structural and metabolic zonation underpins its ability to perform multiple, often disparate, tasks simultaneously.

In multicellular organisms, tissues comprise distinct cell types that execute specialized functions through regulated gene expression (Rouault and Hakim, 2012; Raff, 1996), yet significant cellular heterogeneity exists within populations. This heterogeneity not only fine-tunes normal tissue function but also contributes to variations in disease progression and therapeutic responses (Slyper et al., 2020). In the liver, this complexity is exemplified by the differential roles of various cell types in diseases such as fatty liver disease and fibrosis (Park et al., 2021; Dobie et al., 2019). Liver cells are broadly categorized into parenchymal cells (PCs), primarily hepatocytes and cholangiocytes, which constitute ∼90% of liver mass and are responsible for metabolic and biliary functions (Gissen and Arias, 2015; Jones et al., 2015), and non-parenchymal cells (NPCs). NPCs include liver sinusoidal endothelial cells (LSECs, forming ∼50% of NPCs and the microcirculatory interface) (Aizarani et al., 2019; Gracia-Sancho et al., 2021), kupffer cells (KCs, resident phagocytic macrophages) (Mantovani et al., 2004; Gordon and Taylor, 2005), hepatic stellate cells (HSCs, quiescent vitamin A-storing cells that activate upon injury to produce extracellular matrix and drive fibrosis) (Higashi et al., 2017; De Smet et al., 2021), and recruited immune cells such as monocytes, neutrophils, and natural killer T cells, all dynamically involved in hepatic homeostasis and disease (Kolodziejczyk et al., 2020; Shan et al., 2021). This cellular ecosystem operates on a principle of “parenchymal execution and non-parenchymal regulation” (Ding et al., 2016). To decipher such complexity, scRNA-seq/snRNA-seq have become indispensable. While scRNA-seq profiles whole cells and excels in capturing immune populations, its reliance on tissue dissociation can compromise hepatocyte integrity and bias representation (Andrews et al., 2022). In contrast, snRNA-seq, which analyzes nuclei from fixed or frozen tissues, better preserves the transcriptional state of vulnerable parenchymal cells (PCs), including those from fibrotic livers, offering a complementary approach for comprehensive hepatic profiling (Andrews et al., 2022; Lake et al., 2016). Together, these technologies are revolutionizing our understanding of liver cell heterogeneity, lineage relationships, disease mechanisms, and intercellular crosstalk.

## 2 Overview of single-cell and single-nucleus transcriptomic technologies

scRNA-seq and snRNA-seq are powerful technologies that enable transcriptomic profiling at the resolution of individual cells or nuclei. They provide unparalleled capacity to dissect cellular heterogeneity (MacParland et al., 2018), delineate lineage relationships (Segal et al., 2019), and capture dynamic changes within tissues, thereby profoundly advancing organ, tissue, and cell biology by revealing tissue architecture and function from a foundational single-cell perspective. Unlike traditional bulk sequencing, which averages gene expression across populations, these high-resolution methods uncover the distinct characteristics of each cell. Specifically, scRNA-seq directly captures intracellular mRNA from live cells, making it ideal for discerning differences among cell types and states. Conversely, snRNA-seq isolates and sequences nuclear mRNA, offering a robust alternative for analyzing fixed, frozen, or otherwise difficult-to-dissociate tissue samples, thus opening new avenues to study complex tissues that are not amenable to standard single-cell approaches (Table 1).

**TABLE 1:** Differences between scRNA-seq and snRNA-seq.

Characteristic		scRNA-seq	snRNA-seq
Sample	Preservation method	Fresh samples	Frozen samples
	Cell type	Small cells	Large cells [e.g., neurons (Del-Aguila et al., 2019; Srinivasan et al., 2020), heart (Litvinukova et al., 2020; Vidal et al., 2019), kidney (Lake et al., 2019; O'Sullivan et al., 2019), liver (Aizarani et al., 2019; Rodrigues and Banales, 2021), muscle (Petrany et al., 2020; Lin et al., 2022), adipose tissue (Sarvari et al., 2021; Sun et al., 2020)] or cells difficult to dissociate
Advantages		Suitable for immune cell studies; Detects a more comprehensive gene expression profile.	Applicable to a wider variety of sample types, with relatively simpler workflow; Reduces dissociation-induced transcriptional artifacts; Improves comprehensiveness of captured cell types (Wu et al., 2019).
Disadvantages		Only applicable to fresh tissue samples; Dissociation may lead to loss of specific cell types (van den Brink et al., 2017); Not suitable for large cell types.	Detects fewer genes; Captures fewer immune cells (Slyper et al., 2020; Andrews et al., 2022).

### 2.1 scRNA-seq

scRNA-seq is a high-throughput technology designed to capture genome-wide expression profiles at single-cell resolution, establishing itself as a cornerstone for dissecting cellular heterogeneity (MacParland et al., 2018), lineage relationships (Segal et al., 2019), and gene regulatory networks since its inception in 2009 (Tang et al., 2009). The technology has evolved from early methods like Smart-seq, which provided high sequencing depth at limited throughput, to more scalable platforms such as Drop-seq and 10x Genomics that significantly improved efficiency. Its standard workflow involves single-cell isolation, mRNA capture, reverse transcription, amplification, and sequencing to generate transcriptomic data that reflect the instantaneous state of individual cells, thereby revealing dynamic changes under various physiological or pathological conditions. scRNA-seq has enabled broad applications across biological disciplines. Mapping cell fate trajectories in developmental biology (Segal et al., 2019; Lotto et al., 2020), characterizing immune subset dynamics in immunology (Su T. et al., 2021; Woestemeier et al., 2023), and resolving neuronal and glial diversity in neuroscience (Del-Aguila et al., 2019; Srinivasan et al., 2020). While particularly effective for profiling live cells from fresh tissues with high resolution, the method’s reliance on tissue dissociation can compromise cellular integrity and alter transcriptional profiles (van den Brink et al., 2017), and it may underrepresent certain fragile or structurally complex cell types, such as hepatocytes in fibrotic tissues.

### 2.2 snRNA-seq

snRNA-seq has emerged as a complementary and increasingly vital alternative to scRNA-seq, particularly for tissues that pose challenges for viable single-cell isolation (Rodrigues and Banales, 2021). By directly sequencing nuclear mRNA, snRNA-seq bypasses the need for intact cellular integrity, enabling robust transcriptomic profiling of frozen, fixed, or architecturally complex samples such as fibrotic liver (Rodrigues and Banales, 2021), brain (Del-Aguila et al., 2019; Srinivasan et al., 2020), and heart tissue (Litvinukova et al., 2020; Vidal et al., 2019). This capability has established snRNA-seq as a key tool across diverse fields including neuroscience, oncology, and cardiovascular research (Del-Aguila et al., 2019; Srinivasan et al., 2020; Carlessi et al., 2023; Simonson et al., 2023). Illustrative applications include delineating disease-associated neuronal subpopulations in Alzheimer’s disease (Sadick et al., 2022) and uncovering microenvironment-specific cellular alterations in liver pathologies (MacParland et al., 2018; Aizarani et al., 2019; Armingol et al., 2021; Massalha et al., 2020), thereby providing critical insights for understanding disease mechanisms and identifying potential diagnostic or therapeutic targets.

In summary, scRNA-seq and snRNA-seq offer distinct and often complementary profiles. scRNA-seq is optimal for capturing high-resolution transcriptomes from fresh, dissociable tissues and is especially powerful for immunophenotyping, whereas snRNA-seq extends analysis to preserved or fragile specimens. Recognizing these complementary strengths, an integrated approach, using scRNA-seq to profile fresh samples and snRNA-seq to incorporate archived or difficult-to-dissociate material, is increasingly adopted to construct more comprehensive and reliable cellular atlases (Aizarani et al., 2019). Together, these technologies have become indispensable in modern biomedical research, profoundly advancing our understanding of cellular heterogeneity and gene regulation, and providing a foundational platform for precision medicine. As these methods continue to evolve in accessibility and scalability, their application is poised to expand further, driving more precise mechanistic insights and therapeutic strategies for complex diseases.

### 2.3 Applications of single-cell and single-nucleus transcriptomics in liver tissue

scRNA-seq/snRNA-seq have been extensively applied in liver research to construct comprehensive cellular atlases (Aizarani et al., 2019), delineate developmental trajectories (Dobie et al., 2019; Daemen et al., 2021; Xiong et al., 2019; Krenkel et al., 2019; Krenkel et al., 2020; Seidman et al., 2020), and elucidate the pathogenesis of diverse liver diseases including NAFLD (Woestemeier et al., 2023), hepatitis (Barreby et al., 2022), cirrhosis (Ramachandran et al., 2019), and hepatocellular carcinoma (HCC) (Siegel et al., 2019). These approaches have transformed our understanding of liver biology by moving beyond the averaging limitations of bulk tissue RNA sequencing, which masks the contributions of individual cell types and is unable to resolve rare populations or dynamic shifts in cellular subpopulations during disease (Saviano et al., 2020). In contrast, scRNA-seq and snRNA-seq provide high-resolution maps of tissue heterogeneity, enabling the discovery of novel cell types, the identification of disease-associated differentially expressed genes, the enrichment of pathogenic pathways, and the reconstruction of lineage relationships. This granular view is essential for deciphering the complex, coordinated interactions, such as ligand-receptor signaling and pathway cross-regulation, between PCs and NPCs that define hepatic microenvironments and organ function (Ding et al., 2016), and whose dysregulation underpins disease.

To date, scRNA-seq studies have been pivotal in profiling the human liver in health, cirrhosis, and cancer (MacParland et al., 2018; Aizarani et al., 2019; Armingol et al., 2021; Massalha et al., 2020). The majority of foundational mechanistic insights, however, derive from murine models, where research has focused on decoding NPC crosstalk and characterizing functional hepatocyte subpopulations (Dobie et al., 2019; Daemen et al., 2021; Xiong et al., 2019; Krenkel et al., 2019; Krenkel et al., 2020; Seidman et al., 2020). These studies in classic model organisms have established critical paradigms for liver zonation, immune responses, and fibrotic mechanisms. Notably, the application of these technologies is now expanding into non-model organisms (Table 2), offering comparative perspectives on species-specific adaptations and physiology. When comparing findings, it is crucial to consider the methodological approach. For instance, the pioneering snRNA-seq study in ketotic dairy cows (Feng et al., 2026) successfully captured vulnerable hepatocyte states from frozen tissue, a scenario where scRNA-seq might have failed. In contrast, the detailed immune landscape in HCV-infected tree shrews (Yu et al., 2025) relied on scRNA-seq to fully resolve dynamic immune subsets. Divergences in key findings, such as the specific hepatocyte factor HADHA linked to bovine ketosis (Feng et al., 2026) versus the widespread metabolic reprogramming and interferon response in tree shrew HCV infection (Yu et al., 2025), primarily reflect the distinct pathophysiology being studied. However, a common thread is the revelation of previously unappreciated cellular heterogeneity within the liver parenchyma and immune compartment during disease states, a conclusion that appears robust across species and technologies. Together, the integration of findings from humans, established models, and non-model species through single-cell transcriptomics is refining conserved principles of liver pathobiology while uncovering divergent biological mechanisms, thereby accelerating the translation of discoveries into novel diagnostic and therapeutic strategies.

**TABLE 2:** Application of scRNA-seq and snRNA-seq in the study of liver tissue.

Author	Sequencing methods	Material	Pathway		Correlation analysis	Key findings (cell subtypes/gene)
			Research	Correlation		
Feng et al., (2026)	snRNA-seq	Dairy cow Liver tissue Heathy: n = 1, 8,380 cells Ketosis: n = 1, 25,053 cells	Ketosis	Fatty liver	Metabonomics RNA-seq	Hepatocytes in central venous zone are associated with ketosis; A key factor associated with ketosis: HADHA
Yu et al., (2025)	scRNA-seq snRNA-seq	Tree shrews scRNA-seq: n = 10 snRNA-seq: n = 4	Hepatitis C virus	Liver diseases	RNA-seq	A comprehensive single-cell transcriptomic landscape of HCV-inoculated tree shrew livers
Wu et al., (2024)	scRNA-seq snRNA-seq	Six representative species Liver tissue n = 1/2/3	Liver evolution	—	—	The genetic footprint of the transfer of haem detoxification from the liver to the spleen during vertebrate evolution
Rao et al., (2023)	scRNA-seq scRNA-seq	Pig Liver tissue n = 2/3	Liver development	Metabolic and immune	—	The most comprehensive postnatal liver development single-cell atlas; Demonstrates the metabolic and immune changes across the four age stages
Jiang et al., (2022)	scRNA-seq	Pelodiscus sinensis Liver tissue Heathy: n = 1, 4,443 cells Infection: n = 1, 5495 cells	Infectious diseases	Immune function	scATAC-seq	The hepatic immune landscape of *P. sinensis*
Alvarez et al., (2022)	scRNA-seq snRNA-seq	Human Liver tissue Healthy: n = 1 HCC: n = 3 Adjacent tumor: n = 3	HCC	NAFLD	RNA-seq TCGA	Identified a Prol cell subtype in primary liver cancer
Hundertmark et al., (2022)	scRNA-seq	Mouse Liver tissue	NPCsheterogeneity	NASH	—	Characterized hepatic macrophagepopulations; Identified DEs between healthy and NASH livers
Xu et al., (2022)	scRNA-seq	Human (HCC patients) Liver tissue n = 4, 3,200 cells	Metabolic dysregulation (Energy metabolism)	HCC development	RNA-seq TCGA	Profiled monocytes, T cells, NK cells, Hepatocytes; ADH4, AKR1B10, CEBPZOS, ENO1, FOXN2
Su T. et al., (2021)	scRNA-seq	Mouse Liver tissue Normal: n = 3, 3,248 cells Cirrhosis: n = 3, 4,076 cells	LSECs dysfunction	Liver fibrosis/cirrhosis	—	Defined 6 HCC subgroups; Identified LSEC capillarization genes (e.g., CD34)
Park et al., (2021)	scRNA-seq	Mouse Liver tissue Normal: n = 2, 216 hepatocytes Obese: n = 3, 238 hepatocytes	PC heterogeneity	NAFLD	—	Zone-specific hepatocyte signatures: Periportal (Zone 1) – upregulated gluconeogenic genes; central vein (Zone 3) – upregulated lipid droplet and ketogenic pathway genes
Hao et al., (2020)	scRNA-seq	Human (MacParland et al., 2018) Mouse (Han et al., 2018) liver tissue (database) Human: n = 5, 8,444 cells Mouse: 4,685 cells	Lipid-associated hepatocyte subtypes	Lipid metabolism	GWAS	Periportal hepatocytes (Zone 1)
Xiong et al., (2019)	scRNA-seq	Mouse Liver tissue NPC Normal: n = 3, 17,788 cells Cirrhotic: n = 3, 15,380 cells	NPC heterogeneity	NASH	—	Discovered NASH-associated macrophages (NAMs) and characterized HSC subpopulations.

Author	Sequencing methods	Material	Cell subtypes, gene
Alvarez et al., (2022)	scRNA-seq snRNA-seq	Human Liver tissue n = 4, 73,295 units (29,432 cells; 43,863 nuclei)	PCs: Hepatocytes NPCs: cholangiocytes, liver mesenchymal cells, endothelial cells, intrahepatic monocyte/macrophages, and lymphocytes.
MacParland et al., (2018)	scRNA-seq	Human Liver tissue n = 5, 8,444 cells	Defined 20 liver cell populations (hepatocytes, endothelial, cholangiocytes, HSCs, B cells, T cells, NK-like cells, intrahepatic monocyte/macrophage subsets); Hepatocyte marker: ALB (albumin); Hepatic progenitor marker: AFP (alpha-fetoprotein) (Lotto et al., 2020; Kuhlmann and Peschke, 2006)
Lotto et al., (2020)	scRNA-seq	Mouse n = 15, 45,334 cells	Mapped early liver development, tracing hepatoblast (hepatocyte and cholangiocyte) lineage specification from endoderm and mesoderm origins.

An important complementary dimension to single-cell transcriptomics is spatial resolution. While scRNA-seq and snRNA-seq define cellular states with high molecular granularity, they lose the architectural context that is fundamental to liver function. Spatial transcriptomic technologies (Ben-Moshe and Itzkovitz, 2019; Saviano et al., 2020) now enable mapping of these cell states back onto tissue sections, revealing how periportal versus pericentral positioning shapes hepatocyte transcriptional programs (Halpern et al., 2017), how fibrotic niches organize distinct stromal and immune subsets (Ramachandran et al., 2019), and how tumor-immune interfaces are structured in HCC (Saviano et al., 2020). The convergence of single-cell and spatial approaches is thus poised to transform our understanding of liver biology from a list of cell types to a spatially resolved understanding of tissue function and dysfunction.

### 2.4 Liver-specific methodological considerations

While the general strengths and limitations of scRNA-seq and snRNA-seq are well documented, several liver-specific factors warrant particular attention when designing experiments or interpreting hepatic single-cell datasets.

Standard tissue digestion protocols, necessary for scRNA-seq, can induce rapid transcriptional shifts—including upregulation of stress response genes and downregulation of core metabolic functions—within minutes of disruption (Andrews et al., 2022; van den Brink et al., 2017). Moreover, large polyploid hepatocytes are prone to rupture during dissociation, leading to their systematic underrepresentation in scRNA-seq libraries, a phenomenon exacerbated in diseased livers where PCs accumulate lipid droplets or extracellular matrix encasement (Slyper et al., 2020; Andrews et al., 2022). This technical bias is particularly consequential for studies of metabolic liver diseases such as NAFLD or ketosis, where hepatocyte transcriptional states are central to disease pathogenesis. By contrast, snRNA-seq largely circumvents these issues by isolating nuclei from frozen or fixed tissues, preserving the transcriptional signatures of fragile hepatocytes and enabling unbiased inclusion of PCs across the full spectrum of liver pathology (Andrews et al., 2022; Feng et al., 2026). This methodological distinction explains the increasing adoption of snRNA-seq in studies prioritizing hepatocyte-centric analyses (Feng et al., 2026) and underscores the importance of platform selection based on research objectives. Conversely, snRNA-seq exhibits systematic underrepresentation of immune populations relative to scRNA-seq (Slyper et al., 2020; Andrews et al., 2022). The reasons are multifactorial. Immune cells have smaller nuclei with lower RNA content, their nuclear isolation efficiency is often reduced, and cytoplasmic transcripts critical for functional annotation are partially lost during nuclear preparation. In liver studies, this translates to suboptimal resolution of Kupffer cell heterogeneity, monocyte-derived macrophage subsets, and T-cell dynamics when using snRNA-seq alone (Barreby et al., 2022; Hundertmark et al., 2022). The complementary nature of these technologies is thus particularly evident in the liver. scRNA-seq excels at capturing the rich immune landscape, whereas snRNA-seq ensures faithful representation of parenchymal and stromal cells. Several recent hepatic atlases have successfully leveraged this complementarity by combining both modalities (Aizarani et al., 2019; Andrews et al., 2022; Yu et al., 2025), enabling comprehensive profiling across all liver cell compartments.

The liver field has benefited from a growing number of publicly available single-cell datasets, yet cross-study comparisons remain challenging. Batch effects arising from differences in tissue acquisition (surgical resection vs. biopsy), preservation method (fresh vs. frozen), dissociation protocol (enzyme type, duration), sequencing platform (10x Genomics vs. Smart-seq2), and bioinformatic processing pipelines can obscure biological signals and generate spurious distinctions between studies (Slyper et al., 2020; Saviano et al., 2020). These issues are particularly pronounced when integrating atlases from healthy and diseased livers, where sample handling may differ systematically. For example, cirrhotic liver tissue requires extended enzymatic digestion to release cells from fibrotic matrix, potentially introducing dissociation artifacts that are absent in healthy controls (Su T. et al., 2021; Ramachandran et al., 2019). Similarly, comparisons across species, such as mouse models and human disease, must account for both biological differences and technical variability. The community is increasingly adopting standardized processing frameworks and leveraging computational tools (e.g., Harmony, scVI) to mitigate batch effects, but these challenges underscore the importance of cautious interpretation when synthesizing findings across independent studies.

Collectively, these liver-specific methodological considerations reinforce the value of multi-platform approaches and highlight the need for transparent reporting of experimental parameters. A growing consensus in the field advocates for combined scRNA-seq and snRNA-seq analyses, applied to matched samples when feasible, as the most robust strategy for capturing the full cellular diversity of the liver (Aizarani et al., 2019; Andrews et al., 2022).

## 3 Applications of single-cell and single-nucleus transcriptomics in human liver

The human liver possesses a highly heterogeneous cellular composition and a complex functional architecture. The advent of single-cell and single-nucleus transcriptomic sequencing affords unprecedented opportunities to unravel this complexity. Through the application of these technologies to human liver tissues, researchers can now identify and classify distinct hepatic cell types, elucidate their functional states, and map their intricate interactions. These investigations not only advance the foundational understanding of liver biology in health but also lay the groundwork for dissecting cellular alterations in disease states (MacParland et al., 2018; Aizarani et al., 2019; Armingol et al., 2021; Massalha et al., 2020; Dobie et al., 2019; Daemen et al., 2021; Xiong et al., 2019; Krenkel et al., 2019; Krenkel et al., 2020; Seidman et al., 2020).

### 3.1 Human liver development and cell atlas

The liver’s multifunctionality stems from its complex cellular composition, which derives from multiple developmental lineages. A key focus in liver biology has been to resolve the cellular origins and differentiation pathways during organogenesis. While the parenchyma is known to consist of hepatocytes and bile duct epithelial cells (BECs), the embryonic source of these cells was historically debated. This uncertainty was significantly addressed by scRNA-seq studies of human fetal liver, which identified a hepatobiliary hybrid progenitor (HHyP) population marked by epithelial cell adhesion molecule (EpCAM) expression and possessing a transcriptome distinct from mature hepatocytes or BECs, demonstrating a hybrid hepatic-biliary lineage signature (Segal et al., 2019). Beyond parenchymal origins, scRNA-seq has also illuminated the developmental roots of NPCs. For example, integration of multiple scRNA-seq libraries enabled the reconstruction of a comprehensive lineage map tracing liver cell specification from endodermal and mesodermal precursors (Lotto et al., 2020). In recent years, single-cell and single-nucleus transcriptomic approaches have been systematically deployed to build detailed atlases of the adult human liver. A foundational study by MacParland et al. used scRNA-seq to profile the cellular and immune landscape, identifying 20 discrete cell populations—including hepatocytes, endothelial cells, cholangiocytes, HSCs, and diverse immune subsets—thereby providing a high-resolution reference map of hepatic cellular diversity and the immune microenvironment (MacParland et al., 2018). This work was extended by Aizarani et al., who combined scRNA-seq and snRNA-seq to create a spatially resolved gene expression atlas of the healthy human liver, revealing previously unknown subpopulations of LSECs, KCs, and hepatocytes (HCs), and further delineating heterogeneous epithelial progenitor states within the EPCAM + compartment, such as a TROP2+ intermediate cell cluster (Aizarani et al., 2019). These atlases collectively underscore the remarkable spatial transcriptional heterogeneity of hepatocytes across the lobule, a zonated patterning that is crucial for the liver’s compartmentalized metabolic functions.

### 3.2 Human liver diseases

Single-cell and single-nucleus transcriptomic sequencing have provided critical insights into the cellular and molecular dynamics of prevalent liver diseases, beginning with metabolic disorders such as NAFLD and its progressive form, non-alcoholic steatohepatitis (NASH) (Cohen et al., 2011). Woestemeier et al. combined scRNA-seq with flow cytometry and microbiome sequencing to map liver-infiltrating CD4^+^ T cells in NAFLD/NASH patients, revealing an expansion of multi-cytokine-producing CD4^+^ T cells, particularly a Th17-polarized subset enriched in advanced fibrosis, whose frequency correlated with specific gut microbial communities (Woestemeier et al., 2023). Barreby et al. further performed scRNA-seq on NASH liver biopsies, demonstrating that disease-associated macrophages likely represent activated subsets of resident macrophages and identifying two distinct activated hepatic stellate cell populations linked to fibrosis severity. Their work also highlighted co-regulation of circulating NAFLD markers with GWAS-predicted effector genes in hepatocytes, implicating complement pathway dysregulation in NASH pathogenesis (Barreby et al., 2022). These studies collectively clarify the cellular heterogeneity underlying NAFLD progression from simple steatosis to inflammatory NASH (Diehl and Day, 2017; Samuel and Shulman, 2018) and the potential transition towards cirrhosis, where hepatocytes are replaced by collagen-rich scar tissue (Diehl and Day, 2017; Pais et al., 2016). In advanced liver disease, single-cell technologies have dissected the microenvironmental remodeling in cirrhosis and HCC. Ramachandran et al. profiled over 100,000 single cells from cirrhotic livers, identifying a scar-associated TREM2+CD9^+^ macrophage subset, ACKR1 + PLVAP + endothelial cells restricted to fibrotic niches, and PDGFRα+ collagen-producing mesenchymal cells. Their multi-lineage interaction model revealed active pro-fibrotic pathways including TNFRSF12A, PDGFR, and NOTCH signaling within the scar compartment (Ramachandran et al., 2019). In HCC, scRNA-seq has elucidated tumor heterogeneity and immune microenvironment reprogramming linked to therapy response and prognosis (Ma et al., 2019). Studies have comprehensively characterized the composition, functional states, and developmental trajectories of tumor-infiltrating T cells (Zheng et al., 2017; Zhang et al., 2019). Wang et al. further identified two fibroblast subpopulations marked by Sparcl1 and Gja4, with the Sparcl1+ subset associated with reduced vascular invasion and improved patient survival, offering new insights into stromal cell heterogeneity in HCC (Wang et al., 2021). Together, these applications underscore the power of single-cell transcriptomics in uncovering disease mechanisms and identifying potential therapeutic targets across the spectrum of liver pathology (Siegel et al., 2019).

While the expansion of multi-cytokine-producing CD4^+^ T cells, particularly a Th17-polarized subset, has been highlighted in human NASH livers (Woestemeier et al., 2023), murine models of NASH consistently identify Trem2-expressing disease-associated macrophages (NAMs) as a dominant pathogenic population (Xiong et al., 2019; Hundertmark et al., 2022). This divergence likely reflects both genuine species-specific immune wiring and differences in disease etiology—diet-induced in mice versus multifactorial in humans. Importantly, both lines of evidence converge on the central role of macrophage activation and crosstalk with hepatic stellate cells (HSCs) in driving inflammation and fibrosis. Methodologically, the robust identification of these rare but critical NPC populations in human studies often benefited from snRNA-seq (Andrews et al., 2022; Barreby et al., 2022), which minimizes dissociation-induced loss, whereas mouse studies frequently utilize scRNA-seq for its comprehensive immune cell profiling. This underscores the complementary nature of these technologies in building a complete picture of disease-associated heterogeneity.

## 4 Applications of single-cell and single-nucleus transcriptomics in liver of model animals

Mouse and zebrafish serve as indispensable model organisms for investigating liver biology, owing to their high genetic conservation with humans, experimental tractability, and well-conserved hepatic architecture and function. scRNA-seq/snRNA-seq have been extensively applied in these models to dissect developmental programs, functional zonation, and disease mechanisms with cellular resolution.

In mice, scRNA-seq has enabled the deconstruction of liver lobular organization at unprecedented detail (Gawad et al., 2016). By profiling whole-transcriptome data from individual cells, studies have mapped zonal expression patterns for hundreds of genes, including Hamp, Igfbp2, Mup3, and Cyp8b1, along the portocentral axis (Halpern et al., 2017). This work has elucidated signaling dependencies, such as Wnt signaling for pericentral specification and Ras signaling for periportal identity, and revealed spatially enriched pathways: oxidative phosphorylation genes in periportal regions versus detoxification and bile acid synthesis genes around central veins (Halpern et al., 2017). These findings underscore how scRNA-seq refines our understanding of metabolic zonation, a cornerstone of normal liver physiology. In disease modeling, scRNA-seq has unraveled cell-type-specific responses across a spectrum of liver pathologies. In acute liver injury induced by APAP or TAA, scRNA-seq uncovered previously unknown subpopulations of HSCs, LSECs, KCs, and identified injury-activated cell states (AAs, AAe, AAk) driven by MYC activity, highlighting potential therapeutic targets (Kolodziejczyk et al., 2020). In NAFLD, analysis of >82,000 single cells from mice revealed distinct roles of Kupffer cells versus monocyte-derived macrophages and identified hybrid NPC populations, providing insights into disease progression (Su Q. et al., 2021). NASH studies further identified Trem2-expressing NAMs and delineated HSCs as signaling hubs coordinating vascular and inflammatory responses (Xiong et al., 2019). In fibrosis, scRNA-seq pinpointed collagen-producing central vein-associated HSCs (CaHSCs) and demonstrated that targeting Lpar1 or macrophage YAP signaling attenuates fibrogenesis (Dobie et al., 2019; Qing et al., 2022). In cirrhosis, zonal alterations in LSECs gene expression—including upregulated capillarization markers and downregulated endocytic receptors and vasoregulatory transcription factors (Klf2, Klf4, AP-1)—were mapped, elucidating mechanisms of increased intrahepatic resistance (Su T. et al., 2021).

A comparison of fibrotic mechanisms across models reveals both robust themes and context-dependent specifics. The identification of collagen-producing, central vein-associated HSCs (CaHSCs) as a key profibrotic lineage appears conserved from mouse models (Dobie et al., 2019) to human cirrhosis (Ramachandran et al., 2019). However, the specific signaling hubs identified, such as Lpar1 in mouse HSCs (Dobie et al., 2019) versus PDGFR and NOTCH pathways in the human fibrotic niche (Ramachandran et al., 2019), may vary. These differences could stem from the nature of the injury model (acute vs. chronic), species-specific biology, or the transcriptional snapshot provided by different techniques. The use of scRNA-seq on fresh, dissociated tissue in mouse models (Dobie et al., 2019) excels at capturing activated, matrix-producing cell states, whereas snRNA-seq applied to archived human cirrhotic samples (Ramachandran et al., 2019) was crucial for preserving the complex architecture of the fibrotic scar and its resident cells. Thus, while the principle of HSC activation and mesenchymal cell diversification is a robust cornerstone of liver fibrosis, the precise molecular mediators may be model- or context-dependent, highlighting the need for cross-validation between experimental systems and human pathology.

Beyond mammalian models, zebrafish have emerged as a valuable system for liver research, with scRNA-seq enabling direct comparison to human and mouse biology. Morrison et al. established the first single-cell atlas of the adult zebrafish liver and used a fibrotic model to identify Nrp1 as a mediator of dysregulated Vegf signaling in cholangiocytes, revealing conserved pathways in fibrogenesis (Morrison et al., 2022). Huang et al. subsequently profiled healthy zebrafish liver, identifying ten major cell types and characterizing heterogeneous macrophage and T-cell subsets, along with interactions between immune cells and hepatocytes relevant to NAFLD modeling (Huang et al., 2024). These studies highlight the utility of zebrafish for evolutionary and mechanistic studies of liver disease, providing a complementary platform to rodent models for uncovering conserved and species-specific pathological mechanisms.

## 5 Applications of single-cell and single-nucleus transcriptomics in liver of non-model animals

The application of single-cell transcriptomics has progressively expanded beyond classic model organisms to include non-model species, providing valuable evolutionary, biomedical, and agricultural insights. Non-human primates, such as the *Macaca fascicularis*, offer a highly relevant model due to their close phylogenetic relationship to humans. A comprehensive multi-organ single-cell atlas of the macaque revealed cell-type composition, heterogeneity, and regulatory networks across tissues, demonstrating that macaques share significantly higher similarity with humans than mice in immune gene expression and intercellular communication patterns, establishing them as a superior translational model (Qu et al., 2022). In livestock species, large-scale single-cell atlases have been constructed to bridge biomedical and agricultural research. For pigs, integrated snRNA-seq and scRNA-seq datasets from multiple tissues have been used to build detailed reference maps, systematically characterizing cellular heterogeneity across organs and developmental stages, identifying rare cell populations, and providing resources for precise breeding and human disease modeling (Rao et al., 2023; Wang et al., 2022; Chen et al., 2025a; Chen et al., 2025b). Similarly, a comprehensive single-cell expression atlas covering 59 tissues in dairy cattle has been established, facilitating the study of metabolic traits and lactation biology (Han et al., 2025). Specific studies have focused on the bovine liver, delineating cell subpopulations in key metabolic tissues (Wu et al., 2022; Wu et al., 2023) and identifying hepatocyte subtypes and key factors associated with metabolic disorders such as ketosis (Feng et al., 2026). These resources are pivotal for improving livestock health and productivity.

Beyond mammalian livestock, single-cell transcriptomics is illuminating liver biology across broader evolutionary lineages and specialized disease models. In reptiles, the first single-cell immune atlas of the Chinese soft-shelled turtle liver has been delineated, characterizing immune cell features and responses to bacterial infection, providing a foundational resource for comparative immunology (Jiang et al., 2022). Evolutionary studies leveraging single-cell/nucleus RNA-seq have compared the amphioxus hepatic cecum and vertebrate livers, providing evidence for their homology and demonstrating how gene duplication events contributed to the functional complexity of the vertebrate liver (Wu et al., 2024). Furthermore, non-model organisms are being developed to address specific human disease modeling gaps. For instance, the tree shrew has been employed to study Hepatitis C virus (HCV) infection due to the lack of reliable animal models. Single-cell transcriptomic analysis of HCV-inoculated tree shrew livers revealed widespread interferon-stimulated gene induction across cell types, impaired metabolic pathways in hepatocytes, and identified distinct neutrophil subsets with enhanced NETosis, offering a novel platform for studying HCV pathogenesis and vaccine development (Yu et al., 2025). Collectively, studies in these diverse non-model organisms are enriching our understanding of liver physiology, evolution, and pathology from a comparative perspective, complementing insights gained from traditional model systems.

## 6 Conclusions and future perspectives

**FIGURE 1:**
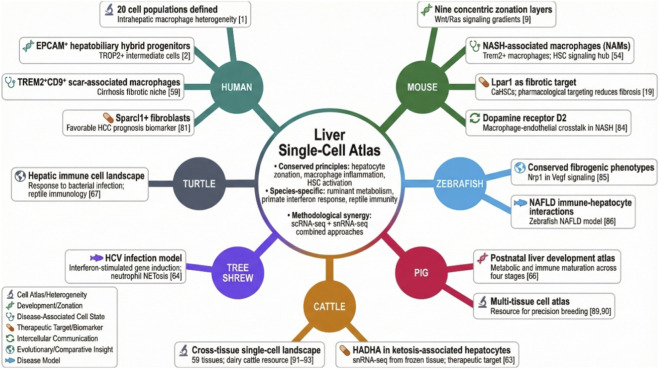
Key discoveries across species: conserved principles and species-specific mechanisms. Representative findings from scRNA-seq and snRNA-seq studies, organized by species and categorized by discovery type. Common themes include the conservation of hepatocyte zonation and macrophage-centered inflammation across mammals, as well as species-specific adaptations such as metabolic specialization in ruminants, unique immune responses to HCV in tree shrews, and comparative immune cell features in reptiles. Collectively, these discoveries illustrate how single-cell technologies are refining conserved principles of liver biology while uncovering divergent mechanisms that inform biomedical, agricultural, and comparative veterinary applications.

The impact of scRNA-seq and snRNA-seq on liver research is unequivocal. These technologies have deconstructed the organ’s cellular complexity, providing unparalleled insights into its development, zonal physiology, and the precise cellular underpinnings of diseases ranging from metabolic dysfunction to cancer. By integrating findings across humans, classic model organisms, and emerging non-model species, a more nuanced and comparative understanding of hepatic biology is being forged. This synthesis not only refines conserved principles of liver function and pathology but also highlights evolutionary adaptations and species-specific mechanisms, enriching both biomedical discovery and translational applications in agriculture and veterinary medicine (Figure 1).

Looking forward, the translational potential of these technologies is only beginning to be realized. Several interconnected directions warrant emphasis. First, multi-omics integration, combining scRNA-seq and snRNA-seq with modalities such as scATAC-seq (Rao et al., 2023), spatial transcriptomics (Ben-Moshe and Itzkovitz, 2019; Saviano et al., 2020), and proteomics, will enable a more holistic view of hepatic cellular states, linking transcriptional profiles to epigenetic regulation and spatial context. Second, clinical translation will depend on validating disease-associated cell states, including Trem2+ macrophages in NASH (Xiong et al., 2019) and scar-associated endothelial cells in cirrhosis (Ramachandran et al., 2019), as biomarkers for patient stratification and therapeutic targeting (Andrews et al., 2022; Saviano et al., 2020). This will require standardized protocols, cost-effective validation methods, and integration with existing clinical parameters. Third, moving from correlation to causation via perturbation models, such as organoids, precision-cut liver slices, and *in vivo* targeting, will be essential to establish functional relevance of candidate pathways identified in atlas studies (Dobie et al., 2019; Qing et al., 2022). Finally, the expansion of single-cell approaches into non-model and agricultural species (Feng et al., 2026; Yu et al., 2025; Wu et al., 2024; Rao et al., 2023) offers unique opportunities for cross-species comparative medicine, enriching both fundamental biology and translational applications across human and veterinary contexts. As these technologies become increasingly scalable and standardized, they are poised to transition from discovery tools to integral components of precision hepatology.
